# Diaphragmatic function is enhanced in fatty and diabetic fatty rats

**DOI:** 10.1371/journal.pone.0174043

**Published:** 2017-03-22

**Authors:** Audrey De Jong, Serge Carreira, Na Na, Aude Carillion, Cheng Jiang, Maud Beuvin, Jean-Marc Lacorte, Dominique Bonnefont-Rousselot, Bruno Riou, Catherine Coirault

**Affiliations:** 1 Sorbonne Universités UPMC Univ Paris 06, UMR INSERM-UPMC 1166, IHU ICAN, Paris, France; 2 Department of Anesthesiology and Critical Care Medicine, Hôpital Pitié-Salpêtrière, Assistance Publique-Hôpitaux de Paris (APHP), Paris, France; 3 Department of Anesthesia and Critical Care B, Hôpital Saint-Eloi, CHU de Montpellier, France; 4 Department of Emergency Medicine and Surgery, Hôpital Pitié-Salpêtrière, APHP, Paris, France; 5 Emergency Department, Zonghnan University Hospital, Wuhan University, Wuhan, The People’s Republic of China; 6 Sorbonne Universités UPMC Univ Paris 06, Inserm, CNRS, Centre de Recherche en Myologie (CRM), GH Pitié Salpêtrière, Paris, France; 7 Department of Endocrinologic and Oncologic Biochemistry, Hôpital Pitié-Salpêtrière, Assistance Publique-Hôpitaux de Paris, Paris, France; 8 Department of Metabolic Biochemistry, Hôpital Pitié-Salpêtrière, APHP, Paris, France; 9 Université Paris Descartes, Faculty of Pharmacy, Paris, France; Institut de Myologie, FRANCE

## Abstract

**Background:**

Obesity is associated with a decrease in mortality in the intensive care unit (ICU) (the "obesity paradox"). We hypothesized that obesity may paradoxically improve diaphragmatic function.

**Methods:**

Diaphragm contractility was prospectively recorded *in vitro* in adult male Zucker lean (control), fatty, and diabetic fatty rats, at rest, after 12h mechanical ventilation and after fatigue. We analyzed diaphragm morphology, cytokines, and protein expression of the protein kinase signaling pathways.

**Results:**

Diaphragm active-force (AF) was higher in fatty (96±7mN.mm^-2^,*P* = 0.02) but not in diabetic fatty rats (90±17mN.mm^-2^) when compared with controls (84±8mN.mm^-2^). Recovery from fatigue was improved in fatty and diabetic fatty groups compared with controls. Ventilator-induced diaphragmatic dysfunction was observed in each group, but AF remained higher in fatty (82±8mN.mm^-2^,*P* = 0.03) compared with controls (70±8mN.mm^-2^). There was neutral lipid droplet accumulation in fatty and diabetic fatty. There were shifts towards a higher cross-sectional-area (CSA) of myosin heavy chain isoforms (MyHC)-2A fibers in fatty and diabetic fatty compared with control rats (*P* = 0.002 and *P*<0.001, respectively) and a smaller CSA of MyHC-2X in fatty compared with diabetic fatty and control rats (*P*<0.001 and *P*<0.001, respectively). The phosphorylated total-protein-kinase-B (pAKT)/AKT ratio was higher in fatty (182±58%,*P* = 0.03), but not in diabetic fatty when compared with controls and monocarboxylate-transporter-1 was higher in diabetic fatty (147±36%,*P* = 0.04), but not in fatty.

**Conclusions:**

Diaphragmatic force is increased in Zucker obese rats before and after mechanical ventilation, and is associated with activation of AKT pathway signaling and complex changes in morphology.

## Background

Obesity and type II diabetes have become a worldwide health concern [1]. Obesity is frequently associated with type II diabetes which further increases the causes of mortality via microvascular and macrovascular complications. In addition obesity increases the risk of atelectasis during mechanical ventilation in the intensive care unit (ICU) [2]. However, obesity and type II diabetes are associated with a lower incidence of acute respiratory distress syndrome (ARDS) [3, 4]. Furthermore, obesity is associated with a decrease in mortality in ICU patients (the "obesity paradox") [5, 6] especially in ARDS patients [7–9].

Recent studies have revealed an interaction between adipocytes and skeletal muscle[10] and this has been associated with skeletal muscle dysfunction [11, 12], insulin resistance [13], and mitochondrial dysfunction [14] in obese and obese diabetic patients. However, the contractile performance of respiratory muscles in obese and obese diabetic patients is, as yet, unknown. In addition, there are only few data on contractile properties of the diaphragm, the main respiratory muscle, in obese [15, 16] and obese diabetic animal models [17] and the published studies reveal conflicting results. Furthermore, prolonged mechanical ventilation induces diaphragmatic dysfunction, a condition known as ventilator-induced diaphragmatic dysfunction (VIDD) [18]. However, no previous studies have assessed VIDD in obese and obese diabetic animals.

The aim of this animal study was to compare diaphragmatic function at rest in Zucker lean (as the control group), Zucker fatty (also known as Zucker obese rats), and Zucker diabetic fatty (also known as Zucker obese diabetic rats) rats *in vitro*. Our experimental model enabled us to precisely assess the mechanical properties of the diaphragm, including contraction, relaxation and contraction-relaxation coupling, and to study diaphragmatic function under various conditions including fatigue, pharmacological stimulation with salbutamol, and prolonged (12 h) mechanical ventilation. We tested the hypothesis that the diaphragm could be paradoxically preserved in fatty and diabetic fatty rats. This may contribute to the improved respiratory prognosis in ICU patients.

## Material and methods

Experiments were conducted in accordance with the Principles of Laboratory Animal Care (NIH publication N0° 86–23, revised 1985) in an authorized laboratory under supervision of authorized researchers (CC, AC). The care and handling of the animals were in accord with National Institutes of Health guidelines. The project had been approved by the Institutional Animal Care and Use Committee through the French Ministry of Higher Education and Research (Comité Régional d’Ethique en Expérimentation Animale Paris-Comité 3, Paris, France, agreement number: 02579.02).

### Animals

Rats were purchased from Charles River (Saint Germain sur l’Arbresle, France) and cared in a labeled housing place (agreement number B-75-13-08) with food and water *ad libitum*. Animals were fed with normal rat chow containing 10% of calories from fat, 67% from carbohydrates, and 23% from proteins, 2.791 kcal/g (A04-10, SAFE, Augy, France). A proportion of these animals were used in another experimental study assessing the β-adrenergic function of the myocardium [19].

Three groups of 15-week old male rats were studied: 1) Zucker lean (control, fa/+) rats (n = 45); 2) Zucker fatty (obese) (fa/fa) rats (n = 15); 3) Zucker diabetic fatty (obese) (fa/fa) rats (n = 20). Because of the obvious morphological differences between groups blinding was not possible. Zucker lean rats were defined as "control", Zucker fatty rats as "fatty" and Zucker diabetic fatty as "diabetic fatty".

### Biological measurements

Total cholesterol and triglycerides concentrations were determined by automated enzymatic methods [20, 21], and glucose concentration was assayed by hexokinase-mediated reaction [22] on a Modular P^®^ chemistry analyzer (Roche Diagnostics, Meylan, France). C-peptide was measured by quantitative ELISA kit provided by Mercodia (Paris, France). Rat leptin and rat total adiponectin levels were measured using kits provided by BioVendor (Eurobio, Courtaboeuf, France) and Alpco (Eurobio) respectively. ELISA was performed according to the manufacturer’s instructions.

### Mechanical characteristics

Rats were anesthetized with sodium pentobarbital (40 mg.kg^-1^ intraperitoneally) and euthanized.

A muscle strip from the ventral costal diaphragm was dissected from the muscle *in situ* and immediately vertically suspended in a 200 ml jacketed reservoir. This reservoir was filled with Krebs-Henseleit bicarbonate buffer solution (NaCl 118 mM, KCl 4.5mM, MgSO_4_ 1.2mM, KH_2_PO_4_ 1.1mM, NaHCO_3_ 25mM, CaCl_2_ 2.5mM and glucose 4.5mM) prepared with highly purified water the hour before. The bathing solution was bubbled with 95% oxygen and 5% carbon dioxide (pH = 7.40) and maintained at 29°C, as previously described.[23] Preparations were field-stimulated (30 V) by using two platinum electrodes. After a 30 min stabilization period at the apex of the length-active isometric force curve (L_*max*_) during which muscle was stimulated by twitches (rectangular-wave pulses of 1 ms duration at 10 pulses/min), diaphragm muscle strips recovered their optimal mechanical performance. Measurements of mechanical variables were made at L_max_ under optimal tetanic stimulation at 50 Hz (10 trains each minute of 300 ms duration, 1 ms rectangular pulses) to obtain maximal strength. Assuming a muscle density of 1.06, cross-sectional area (CSA) was calculated from the ratio of muscle weight to muscle length. All the analyses were made from digital records using an electromagnetic lever system of force and length obtained using specific software, as previously described [24]. Mechanical variables were calculated from three consecutive tetanic contractions preloaded at maximal length (L0). The first contraction was isotonic and loaded with preload only. The extent of shortening (ΔL0), the maximum lengthening velocity (Vr_max_) and the shortening velocity (Vc) were determined from this contraction. The second contraction was fully isometric at L0. The active force, normalized to CSA (AF), the peak of the positive derivative, normalized to CSA (+dF.dt^-1^) and the peak of the negative derivative, normalized per CSA (-dF.dt^-1^) were determined from this fully isometric contraction. The last contraction was abruptly clamped to zero load just after the electrical stimulus, with critical damping in order to slow the first and rapid shortening overshoot resulting from the recoil of series passive elastic components, enabling determination of the maximum shortening velocity (Vmax). As the load the muscle is required to lift decreases, shortening velocity increases until reaching the maximum shortening velocity. Variations in contraction and relaxation must be considered simultaneously to quantify drug-induced changes in lusitropy [25]. Thus, we calculated the ratios Vr/ΔL0 and—dF.dt^-1^/AF, which assess contraction-relaxation coupling in isotonic and isometric conditions, respectively.

### Fatigue, salbutamol and mechanical ventilation

After determination of baseline values, fatigue was induced by repeatedly stimulating the diaphragmatic strip with 75 trains.min^-1^ of 300 ms duration at stimulation frequency of 50 Hz. Stimulation continued until the muscle strip was fatigued to a point where it generated 50–60% of its original tetanic tension measured before the fatigue procedure [26]. Mechanical parameters were recorded just after completion of the fatigue procedure and after 20 min of recovery.

Because β2-adrenergic drugs can increase muscle contraction force mediated by increased calcium release from the sarcoplasmic reticulum [27], the effect of salbutamol was analyzed after measuring baseline mechanical variables. Salbutamol was administered in the Krebs solution at a concentration of 80 μg.L^-1^, as previously described [27]. A period of 15 min for thermoequilibration and diffusion of salbutamol was respected after which contractile mechanical variables were recorded.

In additional groups, rats (n = 20 in control, n = 5 in fatty, n = 9 in diabetic fatty) were ventilated during 12 hours with a volume-preset small animal ventilator (Model 665A; Harvard Apparatus, Holliston, MA). The tidal volume was set at 0.5 ml/100 g body weight, and respiratory rate was set at 55–60 breaths/min. Breathing air was humidified and enriched with oxygen. Ventilator settings and oxygen flow were adjusted to maintain arterial partial pressure of carbon dioxide (PaCO2) between 35 and 40 mmHg and PaO2 between 80 and 100 mmHg. Airway pressure recording was continuously monitored to ascertain complete relaxation of the diaphragm under mechanical ventilation (DP 15–32; Validyne, Northridge, CA). The right carotid artery was cannulated and connected to a pressure transducer to monitor arterial blood pressure and heart rate (blood pressure transducer TD104A; Biopac Systems, Santa Barbara, CA). The tail vein was cannulated for continuous infusion of pentobarbital diluted in isotonic saline (2.5 mg.mL^-1^) at a rate of 1 ml.h^-1^ (Baxter, Deerfield, IL). Additional intraperitoneal anesthesia (pentobarbital, 10 mg) was performed if spontaneous breathing activity was detected. Body temperature was maintained at 37°C throughout the experiment by external warming with a homoeothermic blanket system (Harvard Apparatus).

Animals were euthanized at the end of the 12-h protocol. A muscle strip from the ventral part of the right and left costal diaphragm was carefully dissected *in situ* for the purposes of contractile function study.

### Morphometry

Diaphragm and gastrocnemius cryosections (10 μm- thick) were stained by hematoxylin-eosin (HE) or Oil Red O. Muscle phenotyping was performed according to the expression of myosin heavy chain isoforms (MyHC) by immunofluorescence analysis. Monoclonal antibodies against MyHC isoforms slow MyHC-1 (BA-D5, 1/100), MyHC-2A (SC71, 1/100) and MyHC-2X (6H1, 1/50) were applied at room temperature for 2 hours. All MyHC antibodies were obtained from Developmental Studies Hybridoma Bank (Iowa City, Iowa). Sections were respectively revealed by anti-mouse IgG2b Alexa Fluor 563, anti-mouse IgG1 Alexa Fluor 488 and goat anti-mouse IgM Alexa Fluor 594 (Jackson ImmunoResearchInc., West Grove, PA), and mounted in Mowiol medium. Fluorescence was visualized using a DMRBE Leica microscope equipped with a 40x-oil epifluorescence objective. Sections exposed only to secondary antibodies were used as negative controls and showed no background staining. ImageJ was used to determine muscle fiber type proportion (%), and mean CSA per type. A minimum of 50 fibers/field and 5–8 fields/muscle were measured.

### Immunoblotting

Total proteins were extracted in a Triton 1% buffer (Tris HCl 50mM pH 7.4, NaCl 150mM, 2 mM EDTA, and Triton 1%) with anti-phosphatase/protease inhibitor (Sigma—Aldrich, L'Isle d'Abeau-Chesnes, France). All protein concentrations were determined using Bradford reagent (Bio-Rad, Marne-La-Coquette, France) [28]. After denaturation in Laemmli buffer, a fixed amount of mixed proteins was loaded in each lane of SDS PAGE gel (9–15%). Proteins were separated by electrophoresis in a migration buffer then transferred to a nitrocellulose membrane (Hybond, GE Healthcare, Vélizy, France). After saturation in milk or bovine serum albumin, each membrane was incubated overnight at 4°C with the following primary antibodies: anti- monocarboxylate transporter (MCT) 1 (1/500, AB3540P, Millipore, Billerica, MA), anti- AKT (total protein kinase B, 1/1000, #9272, Cell Signaling Technology, Danvers, MA), anti- pAKT (phosphorylated total protein kinase B, 1/1000, #4058, Cell Signaling Technology), anti- PGC1α (peroxisome proliferator-activated receptor γ coactivator 1-α, 1/1000, ab54481, Abcam, Paris, France), anti- peroxisome proliferator activated receptor (PPAR) α (1/250, ab8934, Abcam), anti- PPARβ/δ (1/200, ab23673, Abcam) [29]. The following day, membranes were washed with tris-buffered saline—Tween (Sigma-Aldrich) and incubated with appropriate secondary antibody. Relative quantification of targeted protein was achieved by fluorescence recording on EthanDIGE reader with an ECL^®^ detection system (GE Healthcare). The monocarboxylate transporter 1 (MCT1) protein was detected at 40 kDa, AKT at 60 kDa, pAKT at 60 kDa, PGC1α at 105 kDa, PPARα at 52 kDa and PPARβ/δ at 50 kDa. All Western blot experiments were quantified using Image J software (NIH, Bethesda, MA) and normalized against GAPDH (37 kDa, Abcam) for PGC1α, PPARα and PPARβ/δ, and against β-actin (42 kDa, Abcam) for AKT, pAKT, and MCT1, ensuring no variation in protein gel loading.

### Quantitative Polymerase Chain Reaction (PCR)

RNeasy mini kit (Qiagen, Courtaboeuf, France) was used to prepare total RNA. Proteinase K step was incorporated according to the manufacturer instructions. For reverse transcription and quantitative RT-PCR, Superscript III (Life technologies, Saint-Aubin, France) with random primers was used for cDNA generation and SYBR Green PCR Master Mix was used according to manufacturer instructions. Experiments were performed on Light Cycler 480 System (Roche Diagnostics) with each sample performed in triplicate. mRNA expression of interleukin (IL)-6 was analyzed in the diaphragm at rest and after mechanical ventilation. Acidic ribosomal protein gene was selected as the housekeeping gene. Fold-change values of cytokine-specific data in comparison to independent experiments are given to indicate relative expression levels of cytokines.

### Statistical analysis

Quantitative variables were expressed as means ± standard deviation (SD) and qualitative variables were expressed as numbers (percentage). Comparison of means for unpaired data was performed using analysis of variance and Tukey's test for multiple comparisons. Comparison of means for paired data was performed using repeated-measures analysis of variance and Tukey's test for multiple comparisons. Comparison of percentages was performed using a Chi square test with Bonferroni correction. Comparison of CSA fibers distribution was performed using Wilcoxon’s test. Assuming a baseline value of force of 80 ± 9 mN.mm^-2^ [26], an alpha risk of 0.05 and a beta risk of 0.20, we determined that a sample size of n = 10 per group enables us to detect a 15% increase in force at rest in fatty rats. All *p* values were two-tailed and a *p* value of less than 0.05 was considered significant. Statistical analysis was performed using SAS software version 9.3 (SAS Institute; Cary, NC).

## Results

Table 1 presents the main baseline characteristics of the control, fatty and diabetic fatty rats.

**Table 1:** General characteristics and main mechanical variables at baseline and after 12h of mechanical ventilation of Zucker control, fatty, and diabetic fatty rats.

Variable	Control	Fatty	Diabetic fatty
***At baseline***			
**General characteristics (no. of rats)**	(n = 45)	(n = 15)	(n = 20)
Body weight (g), mean ± SD	341 ± 19	492 ± 28*†	356 ± 20*
**Biological Measurement (no. of rats)**	(n = 10)	(n = 10)	(n = 9)
Blood glucose level (mmol/l), mean ± SD	8.6 ± 1.6	8.6 ± 1.2†	36.1 ± 4.9*
Total cholesterol (mmol/l), mean ± SD	2.58±0.22	6.26±0.71*†	4.64±0.42*
Triglycerides (mmol/l), mean ± SD	1.39±0.69	3.73±1.38*	3.00±1.59*
	(n = 8)	(n = 9)	(n = 9)
Leptin (μg.L^-1^), mean ± SD	5.2 ± 1.9	61 ± 11.8*†	10 ± 1.42*
Adiponectin (mg.L^-1^), mean ± SD	3.2 ± 0.4	3.3 ± 0.5	4.3 ± 1.0
C peptide (μmol.L^-1^), mean ± SD	1.29 ± 0.51	6.59 ± 2.25*†	1.85 ± 0.53*
**Diaphragm characteristics (no. of rats)**	(n = 25)	(n = 10)	(n = 11)
*Diaphragm strip*			
Section (mm²), mean ± SD	1.3 ± 0.1	1.3 ± 0.1	1.3 ± 0.2
L0 (mm), mean ± SD	11.8 ± 1.9	11.0 ± 1.8	11.6 ± 2.5
*Contraction*			
ΔL0 (%L0), mean ± SD	26 ± 7	30 ± 11	31 ± 12
V_max_ (L0.s^-1^), mean ± SD	6.3 ± 0.7	6.9 ± 1.2	6.8 ± 1.2
Vc (L0.s^-1^), mean ± SD	3.5 ± 0.6	3.2 ± 0.6	3.3 ± 0.6
AF (mN.mm^-2^), mean ± SD	84± 7	96± 7*	90± 17
+dF.dt^-1^ (mN.mm^-2^.s^-1^), mean ± SD	882 ± 162	1,358 ± 583*†	915 ± 261
*Relaxation*			
Vr_max_ (L0.s^-1^), mean ± SD	-9.1 ± 2.1	-8.5 ± 2.6	-8.3 ± 3.1
-dF.dt^-1^ (mN.mm^-2^.s^-1^), mean ± SD	-1,468 ± 369	-2,490 ± 1174*†	-1,605 ± 772
*Contraction-Relaxation coupling*			
Vr/ ΔL_max (_L0.s^-1^.%L0_),_ mean ± SD	0.36 ± 0.09	0.29 ± 0.07	0.28 ± 0.07*
-dF.dt^-1^/ AF (s^-1^), mean ± SD	17.6 ± 4.1	25.6 ± 11.0*†	17.3 ± 5.8
***After mechanical ventilation***			
**Diaphragm characteristics (no. of rats)**	(n = 20)	(n = 5)	(n = 9)
*Diaphragm strip*			
Section (mm²), mean ± SD	1.4 ± 0.2	1.4 ± 0.2	1.4 ± 0.2
L0 (mm), mean ± SD	10.8 ± 1.4	10.4 ± 1.5	11.6 ± 1.4
*Contraction*			
ΔL0 (%L0), mean ± SD	27 ± 10	28 ± 8	22 ± 7
V_max_ (L0.s^-1^), mean ± SD	6.3 ± 0.8	6.6± 0.6	5.6 ± 0.7
Vc (L0.s^-1^), mean ± SD	3.2 ± 0.6	3.4 ± 0.3†	2.6 ± 0.6
AF (mN.mm^-2^), mean ± SD	70± 8	82± 8*	74± 10
+dF.dt^-1^ (mN.mm^-2^.s^-1^), mean ± SD	739 ± 127	950 ± 442	745 ± 104
*Relaxation*			
Vr_max_ (L0.s^-1^), mean ± SD	-9.8 ± 2.1	-8.8 ± 3.1	-7.7 ± 1.5*
-dF.dt^-1^ (mN.mm^-2^.s^-1^), mean ± SD	-1454 ± 469	-1792 ± 838	-1355 ± 522
*Contraction-Relaxation coupling*			
Vr/ ΔL0 _(_L0.s^-1^.%L0_),_ mean ± SD	0.39 ± 0.11	0.32 ± 0.08	0.37 ± 0.09
-dF.dt^-1^/ AF (.s^-1^), mean ± SD	20.6 ± 5.5	21.6 ± 9.1	18.2 ± 5.9

*: *P* < 0.05 versus control group;

^†^: *P* < 0.05 versus diabetic fatty

ΔL_max_ = maximal extent of shortening; V_max_ = maximal unloaded shortening velocity; Vc = maximum shortening velocity; AF = maximal isometric active force normalized per cross-sectional area; +dF/dt = peak of the positive force derivative normalized per cross-sectional area; Vr_max_ = maximal lengthening velocity; -dF/dt = peak of the negative force derivative normalized per cross-sectional area. Biological measurements were already reported in a previous study [19].

### Mechanical performances

**Fig 1:**
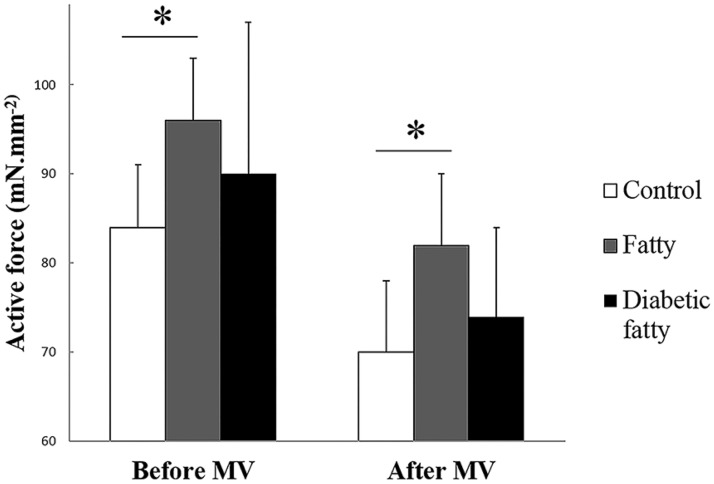
Isometric active force normalized for cross—sectional area in control (n = 25), fatty (n = 10) rats and diabetic fatty (n = 11) rats, at baseline and after 12 hours of mechanical ventilation. Values are expressed as mean percent of baseline ± SD.*: *P* < 0.05 versus baseline.

Fatty rats exhibited improved diaphragm contractile performance, as shown by significantly higher AF and +dF/dT in fatty than controls (*p* = 0.02) (Table 1, Fig 1). There were no significant differences regarding AF and +dF/dT between diabetic fatty and control groups (*p* = 0.21) (Table 1, Fig 1). Shortening performance, as shown by V_max_ and ΔL0 did not significantly differ between groups (Table 1). Moreover, -dF.dt^-1^ and the -dF.dt^-1^/AF ratio, which tested the relaxation phase and the contraction-relaxation coupling under isometric conditions respectively, were higher in fatty when compared to control and diabetic fatty groups (Table 1), thus attesting to faster relaxation coupling under isometry in fatty diaphragms.

**Fig 2:**
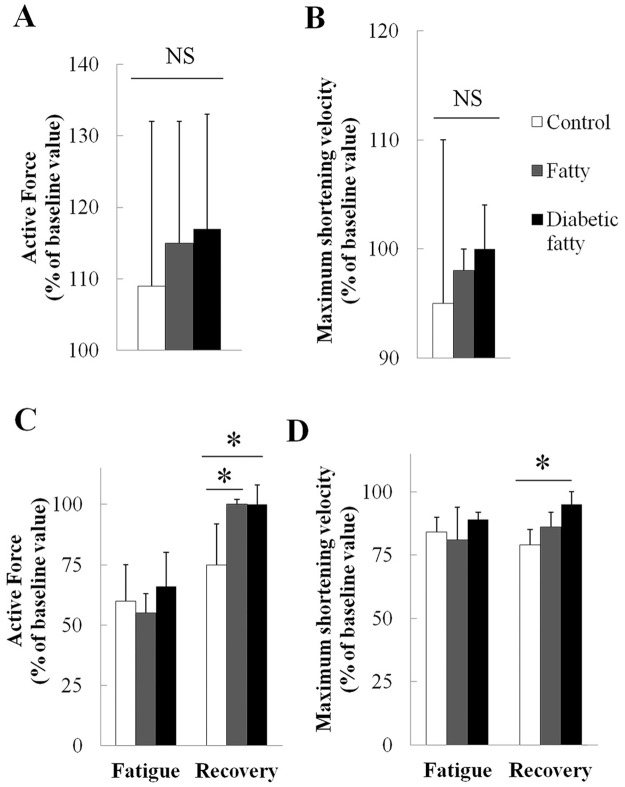
Evolution of isometric active force (AF, panel A) normalized for cross—sectional area and maximum shortening velocity (V_max_, panel B) after salbutamol in control (n = 11), fatty (n = 6) rats and diabetic fatty (n = 4) rats and of AF (panel C) normalized for cross—sectional area and V_max_ (panel D) during fatigue and early recovery phase of fatigue in control (n = 14), fatty (n = 4) rats and diabetic fatty (n = 7) rats. Values are expressed as mean percent of baseline ± SD.*: *P* < 0.05 versus baseline. NS = not significant. The y axis in graphs 2A and 2B does not start at zero. ^1^The differences are not significant between groups.

After treatment with salbutamol, AF (Fig 2A) and V_max_ (Fig 2B) percentages of baseline values did not significantly differ between groups (*p* = 0.71 and *p* = 0.69 respectively). Compared to baseline, treatment with salbutamol induced significant increase in AF (*p* = 0.01) (Fig 2A) but not in V_max_ (*p* = 0.12) (Fig 2B).

**Fig 3:**
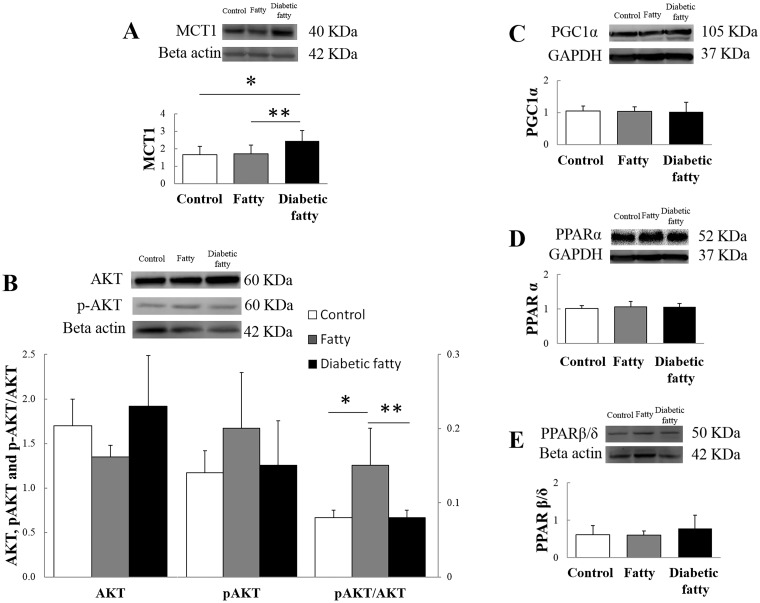
Representative western blots reflecting Monocarboxylate Transporter (MCT) 1 (panel A), total protein kinase B (AKT, left y-axis), phosphorylated (P)-AKT (right y-axis) and ratio P-AKT/AKT (right y-axis, panel B), PGC1 α (peroxisome proliferator-activated receptor γ coactivator 1-α) (panel C), peroxisome proliferator activated receptor (PPAR) α (panel D), PPAR β/δ (panel E) protein expression in Zucker control, fatty, and diabetic fatty rats (n = 7 in each group). Values are expressed as mean ± SD. *P < 0.05 versus control; **: *P* < 0.05 versus diabetic fatty. GAPDH = glyceraldehyde 3-phosphate dehydrogenase.

The fatigue protocol was continued until equivalent values of AF and V_max_ were reached in each group (see methods). Twenty minutes after the end of the fatigue protocol, AF (% of baseline value) recovery was significantly higher in both fatty (*P* = 0.007) and diabetic fatty (*P* = 0.002) than in control groups. V_max_ (% of baseline value) was significantly higher in diabetic fatty (*P* < 0.001) but not in fatty rats (*P* = 0.09) when compared with controls (Fig 2C and 2D). Because lactic acid accumulation has been involved in muscle fatigue [30] we analyzed the protein expression of the lactate transporter MCT1 and found that MCT1 was significantly higher in diabetic fatty (147 ± 36%, *P* = 0.04), but not in fatty (103 ± 29%, *P* = 0.99), when compared with controls (Fig 3A).

### Muscle morphometry and signaling pathway

**Fig 4:**
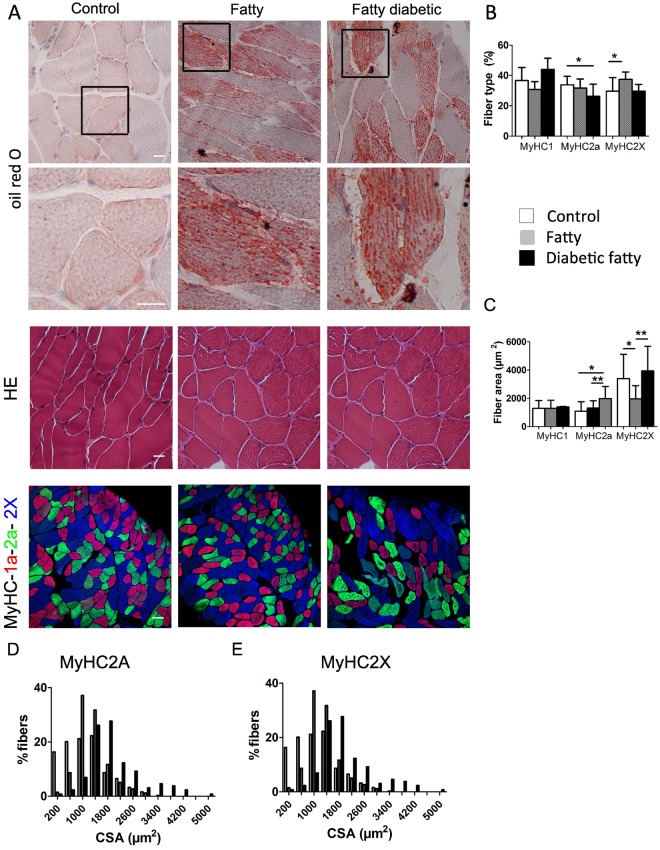
Morphometric study of diaphragm in control (n = 5), fatty (n = 5) and diabetic fatty (n = 5) rats at rest. **Panel A: Histological characterization of diaphragm using Oil red O, hematein/eosin (bar = 20 μm) and Myosin Heavy Chain (MyHC) isoforms (bar = 50 μm) stainings. Zoom-in of Oil red O stainings are presented in lower panels. Panel B: fiber type composition (%). Panel C: fiber type area (μm²). Panel D: MyHC2A fiber distribution by cross sectional area. Panel E: MyHC2X fiber distribution by cross sectional area.** Values are expressed as mean ± SD or percentages ± SD. *: *P* < 0.05 versus control; **: *P* < 0.05 versus diabetic fatty. HE = hematoxylin-eosin coloration.

Control diaphragms were negative for neutral lipid accumulation. In contrast, oil red O staining revealed substantial neutral lipid droplet accumulation in fatty and diabetic fatty rats (Fig 4A). Importantly, there was no evidence of intramuscular adipocyte accumulation in the three groups and HES staining did not evidence morphologic difference between groups. Compared with controls, fatty rats exhibited a significant higher percentage of MyHC-2X fibers (149 ± 25%, *P* = 0.006, Fig 4B), and were associated with significant reduction of the CSA of MyHC-2X fibers (59 ± 13%, *P* = 0.006, Fig 4C). Diabetic fatty rats exhibited a significant lower percentage of MyHC-2A fibers (64 ± 28%, *P* = 0.04, Fig 4B), but with significant larger MyHC-2A fiber CSA (160 ± 28%, *P* = 0.004, Fig 4C). There was a shift towards higher CSA of MyHC-2A fibers in both the fatty and diabetic fatty groups compared with controls (*P* = 0.002 and *P* < 0.001, respectively, Fig 4D) and a shift towards smaller CSA of MyHC-2X in fatty compared with diabetic fatty and control rats (*P* < 0.001 and *P* < 0.001, respectively, Fig 4E). No significant difference in the CSA of MyHC-1 fibers were observed between groups (Fig 4C).

To determine whether these morphological differences between groups were specific for the diaphragm, we also analyzed neutral lipid accumulation and the fiber type characteristics in a fast-type muscle of the limb, namely the gastrocnemius (S1 Fig). Oil-red O staining revealed substantial neutral lipid droplet accumulation in the gastrocnemius of fatty, but not of diabetic fatty rats (S1 Fig). In addition, fatty and diabetic fatty rats exhibited significantly smaller MyHC-2X fibers (respectively 59 ± 23%, *P* < 0.001, and 58 ± 25%, *P* < 0.001) (S1 Fig) compared with controls.

We then asked whether changes in fiber CSA and muscle performance can be associated with changes in the main signaling pathway related to muscle growth, i.e. the AKT signaling pathway. The pAKT/AKT ratio was significantly higher in fatty (182 ± 58%, *P* = 0.03), but not in diabetic fatty rats (95 ± 16%, *P* = 0.99), when compared with controls (Fig 3B). Finally, we considered whether potential changes in nuclear receptors involved in the regulation of muscle fatty acid metabolism may contribute to explain the improved muscle performance in fatty rats. No significant changes were observed regarding the protein expression of PGC1α (*P* = 0.97) (Fig 3C), PPARα (*P* = 0.60) (Fig 3D) and PPARβ/θ (*P* = 0.81) (Fig 3E).

### Effect of mechanical ventilation

After 12h of mechanical ventilation, AF was significantly reduced in each group when compared with AF before mechanical ventilation (83 ± 10% of AF at rest in control, *P* < 0.001, 85 ± 8% in the fatty, *P* = 0.007, 82 ± 11% in the diabetic fatty groups, *P* < 0.001) (Table 1). The reduction in AF after mechanical ventilation did not significantly differ between groups (*P* = 0.87). As before ventilation, AF was significantly higher in fatty than in control after mechanical ventilation (*P* = 0.03) (Fig 1, Table 1). After mechanical ventilation, there was a significant reduction in the CSA affecting Type 1 fibers only (S2A Fig), in lean (*P* < 0.001), fatty (*P* = 0.007) and diabetic fatty (*P* < 0.001) (S2B Fig).

### Diaphragm cytokine expression

Because increased inflammatory state in muscle has been associated with muscle dysfunction, diaphragm cytokine expression was analyzed both at baseline and after mechanical ventilation (S3 Fig). At baseline, the diaphragm mRNA expression of TNFα did not differ between groups. After mechanical ventilation, TNFα was significantly increased in diabetic fatty group (*P* < 0.05) but not in lean and fatty groups compared with baseline values. At baseline, the diaphragm mRNA expression of IL-6 did not differ between groups (S3 Fig). After mechanical ventilation, IL-6 was significantly increased in the 3 groups compared with corresponding baseline values (*P* < 0.05, S3 Fig). At baseline, IL-1β was significantly higher in fatty than in diabetic fatty group (*P* < 0.05, S3 Fig). After mechanical ventilation, IL-1β was significantly increased in lean and diabetic fatty groups (*P* < 0.05, S3 Fig) but not in fatty compared with baseline values.

## Discussion

In the present study, we observed enhanced diaphragmatic function in fatty, but not in diabetic fatty rats. Moreover, in both fatty and diabetic fatty rats, recovery from fatigue was improved. Mechanical ventilation was associated with a consistent reduction in the diaphragm performance across the groups, reflecting VIDD. As observed before mechanical ventilation, AF remained higher in fatty than in control after mechanical ventilation. These mechanical differences were associated with complex morphometric changes between groups and activation of AKT signaling pathway.

Diaphragmatic function has been largely studied in various physiological [31] and pathological conditions [23, 32], but very few studies have assessed diaphragmatic function in an obese population. Van Lunteren et al. [16] did not report a significant difference in diaphragmatic force between fatty and control rats. Farkas et al. [15] have found that overall diaphragm thickness is selectively greater in obese animals. In the present study, the mechanical variables were consistent with an enhanced diaphragmatic performance in fatty rats (Table 1). Consistently, immunoblotting analysis revealed a higher pAKT/AKT ratio in the fatty group when compared with control and diabetic fatty groups (Fig 3B). Activation of AKT signaling pathway, known to be associated with increased skeletal muscle mass and force [33] may explain the higher AF observed in fatty diaphragms. Moreover, activation of the AKT signaling pathway was associated with complex changes in type and CSA of fibers, the increase in type IIA fiber CSA being associated with a shift towards smaller type IIX fibers (Fig 4C).

Recovery from fatigue was enhanced in fatty and diabetic fatty rats. These results are consistent with the increased resistance to fatigue previously observed in type I diabetic rats [26]. PPARβ/δ, expressed mostly in heart and skeletal muscle, is involved in the fast-to-slow fiber shift induced by endurance training [34]. In accordance with histological results, PPARβ/δ was unchanged in the present study, consistently with the lack of modification of PGC1α, also involved in the regulation of fatty acid metabolism [35]. However, immunoblotting analysis revealed that lactate transporter MCT1 was significantly increased in the diabetic fatty group when compared with control and obese groups, which may explain the better recovery from fatigue in diabetic fatty rats, through a better elimination of lactate [36]. In addition, the improved recovery of AF after fatigue in fatty and diabetic fatty rats might be explained by the shift to higher CSA in type IIA fibers (Fig 4) and by the activation of AKT signaling pathway in fatty rats (Fig 3B).

To our knowledge, this is the first study to assess diaphragmatic function of fatty and diabetic fatty rats after 12 hours of mechanical ventilation. As observed in baseline conditions, AF of diaphragm was higher in fatty than in control after mechanical ventilation. AF was consistently reduced across groups, ranging from 82% to 85% of AF at rest, reflecting a VIDD. The loss in specific force is multifactorial, and probably related to intrinsic changes in regulation of muscle contraction.

The present study may allow a better understanding of the "obesity paradox" in the ICU [37]. Obesity and mortality in ICU are inversely associated as shown by recent studies [38, 39] and meta-analyses [5, 6, 40]. In particular, ARDS obese patients, in whom diaphragmatic function is challenging, have a lower mortality risk when compared with non-obese patients [7–9]. Given that diaphragm dysfunction following ICU admission has been associated with a poorer prognosis [41] one could hypothesize from our results that the improved diaphragmatic function in obese patients admitted to ICU could explain improved mortality.

The following limitations of the study should be considered when assessing the clinical relevance of our results. First, this *in vitro* study only dealt with intrinsic diaphragmatic contractility and the relevance of the leptin receptor-deficient rat model for Type 2 diabetes mellitus and obesity is still a matter of discussion [42]. A high-fat diet group could have been of interest. Our results cannot be directly applied to humans without further human studies [43]. However, we used one of the most commonly used animal models of obesity [27, 43] and VIDD [44, 45] and the results were consistent throughout the different conditions analysed. Second, the ventilatory capacities measured by plethysmography were not assessed. However, plethysmography measures would probably be disappointing, because respiratory physiology in obese patients is known to be affected both at rest and during exercise. Ventilatory capacities are decreased in obese patients compared to non-obese patients, with reduction in expiratory reserve volume, functional residual capacity, respiratory system compliance and impaired respiratory system mechanics. These changes produce a restrictive ventilatory defect, expiratory flow limitation and the development of intrinsic positive end expiratory pressure [46–49]. These modifications associated with increased oxygen consumption result in an increase of work of breathing in obese patients compared to non-obese patients. The increased diaphragmatic force could be an adaption to this increased work of breathing, well demonstrated in the literature in obese patients [48–51]. The muscle benefits observed in fatty rats are probably not an intrinsic primary event responsible of a better ventilation. On the contrary, the muscle benefits would be the consequence of an increased excitation-contraction coupling derived from an increased activity of neuronal pattern that controls ventilation, because of an adaptive consequence of respiratory physiology changes in the obese rats. Third, AKT is a kinase, cornerstone of inflammatory, growth and metabolic intracellular pathways. Other proteins of the mammalian target of rapamycin (mTOR) cascade were not assessed. Fourth, the observed changes of muscle fibers are complex. Therefore, the increase of pAKT/AKT in fatty rats compared to controls is but one of numerous hypotheses to explain the increase in mass and force. Fifth, although we used the most common model for the study of VIDD, this model suffers from significant limitations due to early mortality and animals rarely survive more than a day when ventilated. This might add a significant confounding factor related to a deteriorating preparation not primarily related to the effects of mechanical ventilation and VIDD.

In conclusion, we report improved diaphragmatic contractile performance in Zucker obese rats and an improved recovery from fatigue in Zucker obese and obese diabetic rats, which may be related to activation of AKT signaling pathway and complex diaphragmatic modifications. As observed in baseline conditions, the mechanical performance of the diaphragm after mechanical ventilation was better in obese rats than in control rats. These results may at least partly contribute to the explanation of the obesity paradox which has been described in patients admitted to the ICU.

## Supporting information

S1 Fig Morphometric study of gastrocnemius muscle in control (n = 5), fatty (n = 5) and diabetic fatty (n = 5) rats. Panel A: gastrocnemius hematoxylin-eosin and Oil red O staining (bar = 10 μm). Panel B: fiber type area (μm²). Values are expressed as mean ± SD or percentages ± SD. *: *P* < 0.05 versus control. HE = hematoxylin-eosin coloration.
(TIF)

S2 Fig Morphometric study of diaphragm in control (n = 5), fatty (n = 5) and diabetic fatty (n = 5) rats after mechanical ventilation. Panel A: Hematoxylin-eosin and Oil red O staining (bar = 10 μm). Panel B: fiber type composition (%). Panel C: fiber type area (μm²). Values are expressed as mean ± SD or percentages ± SD. *: *P* < 0.05 versus Control. HE = hematoxylin-eosin coloration.
(TIF)

S3 Fig Diaphragm cytokine expression in control (n = 9), fatty (n = 9) and diabetic fatty (n = 9) at baseline and after mechanical ventilation. Panel A: tumor necrosis factor (TNF) α messenger ribonucleic acid (mRNA) (fold change). Panel B: interleukin (IL)-6 mRNA (fold change). Panel C: IL-1β mRNA (fold change). *: *P* < 0.05 versus Control; **: *P* < 0.05 versus diabetic fatty. Values are expressed as mean ± SD. n = 9 corresponds to 3 triplicates.
(TIF)
