# Supplementary figures and images for: Diaphragmatic function is enhanced in fatty and diabetic fatty rats
Source: PLoS One. 2017 Mar 22;12(3):e0174043. doi: 10.1371/journal.pone.0174043 (PMC5362060; doi:10.1371/journal.pone.0174043)

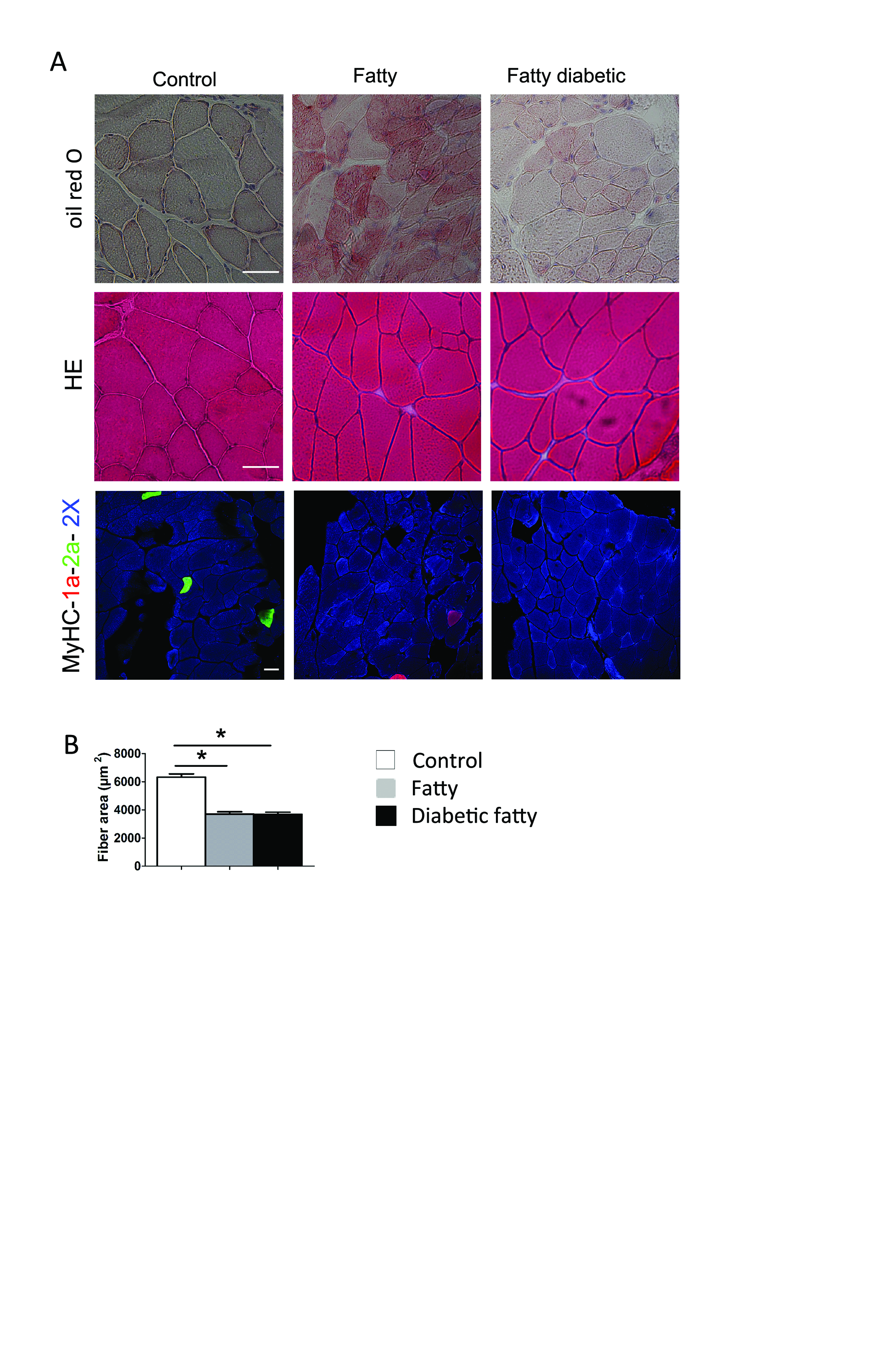

Supplement: S1 Fig — Panel A: gastrocnemius hematoxylin-eosin and Oil red O staining (bar = 10 μm). Panel B: fiber type area (μm²). Values are expressed as mean ± SD or percentages ± SD. *: P < 0.05 versus control. HE = hematoxylin-eosin coloration. (TIF) [file pone.0174043.s001.tif]

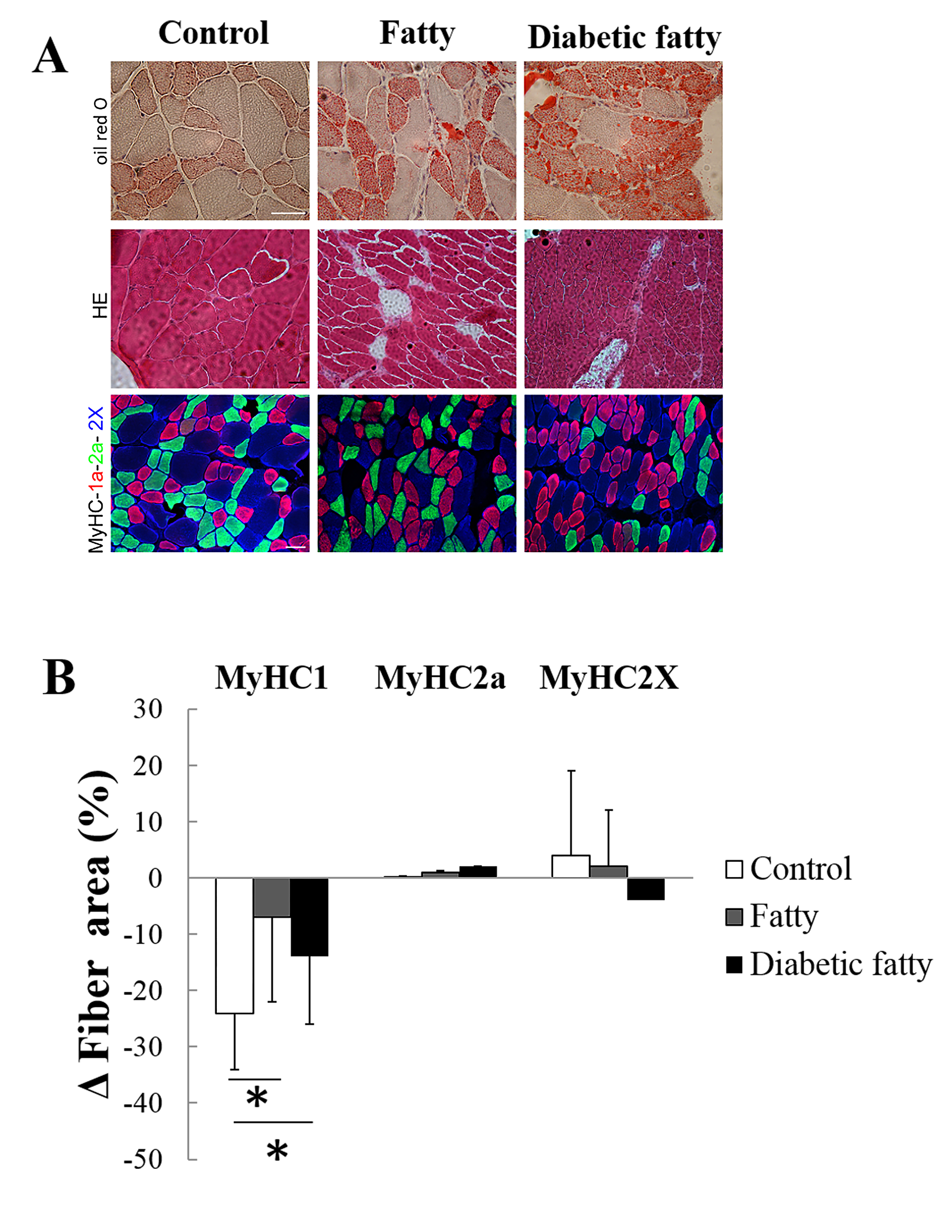

Supplement: S2 Fig — Panel A: Hematoxylin-eosin and Oil red O staining (bar = 10 μm). Panel B: fiber type composition (%). Panel C: fiber type area (μm²). Values are expressed as mean ± SD or percentages ± SD. *: P < 0.05 versus Control. HE = hematoxylin-eosin coloration. (TIF) [file pone.0174043.s002.tif]

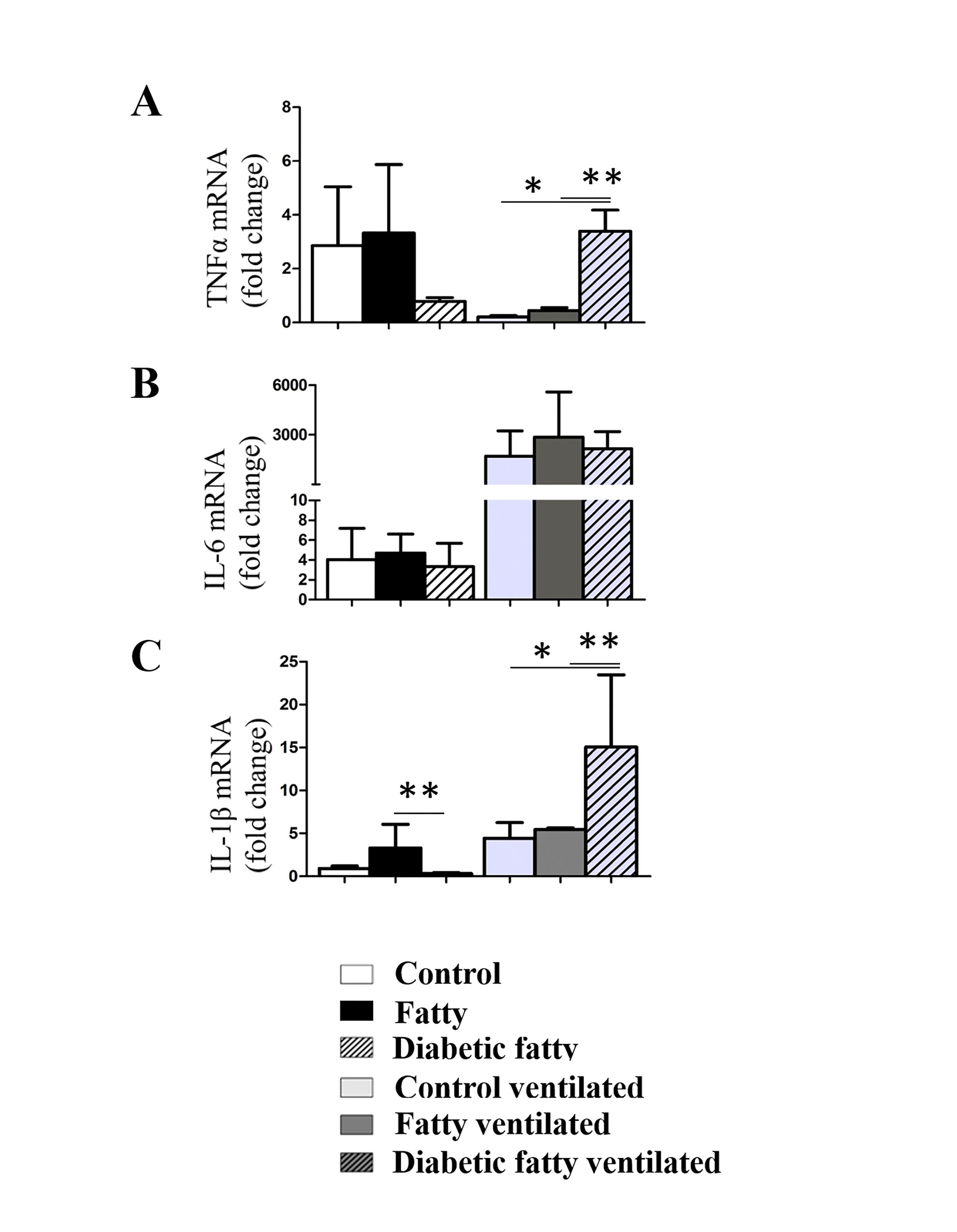

Supplement: S3 Fig — Panel A: tumor necrosis factor (TNF) α messenger ribonucleic acid (mRNA) (fold change). Panel B: interleukin (IL)-6 mRNA (fold change). Panel C: IL-1β mRNA (fold change). *: P < 0.05 versus Control; **: P < 0.05 versus diabetic fatty. Values are expressed as mean ± SD. n = 9 corresponds to 3 triplicates. (TIF) [file pone.0174043.s003.tif]
